# Supplementary figures and images for: Prognostic Role of Survivin and Macrophage Infiltration Quantified on Protein and mRNA Level in Molecular Subtypes Determined by RT-qPCR of KRT5, KRT20, and ERBB2 in Muscle-Invasive Bladder Cancer Treated by Adjuvant Chemotherapy
Source: Int J Mol Sci. 2020 Oct 8;21(19):7420. doi: 10.3390/ijms21197420 (PMC7582791; doi:10.3390/ijms21197420)

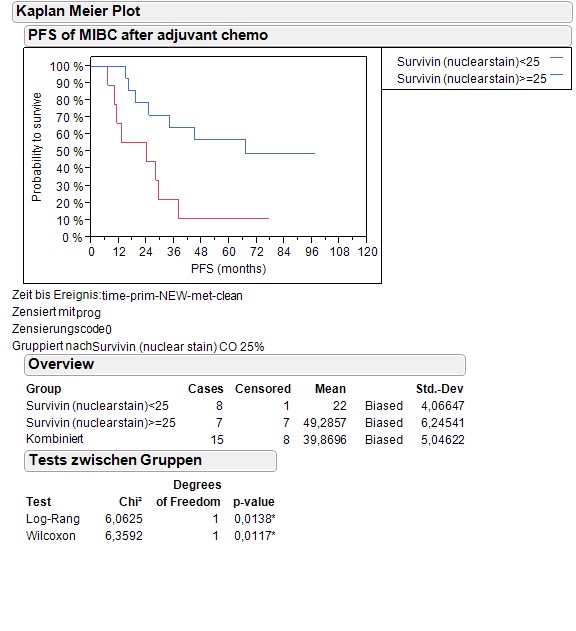

Supplement: Supplementary file 1 [file ijms-21-07420-s001.zip › supplementary figures Figure S1-S13/figS01.jpg]

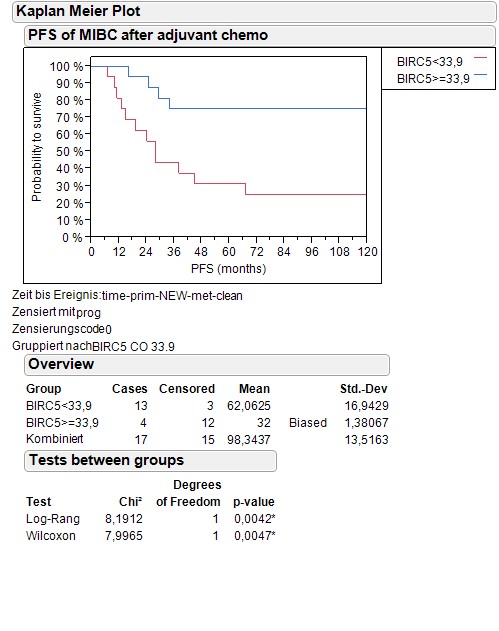

Supplement: Supplementary file 1 [file ijms-21-07420-s001.zip › supplementary figures Figure S1-S13/figS02.jpg]

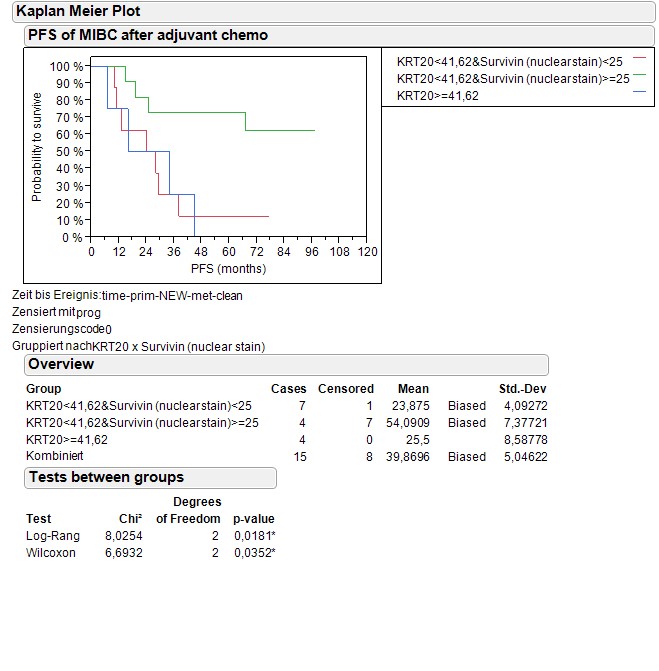

Supplement: Supplementary file 1 [file ijms-21-07420-s001.zip › supplementary figures Figure S1-S13/figS03.jpg]

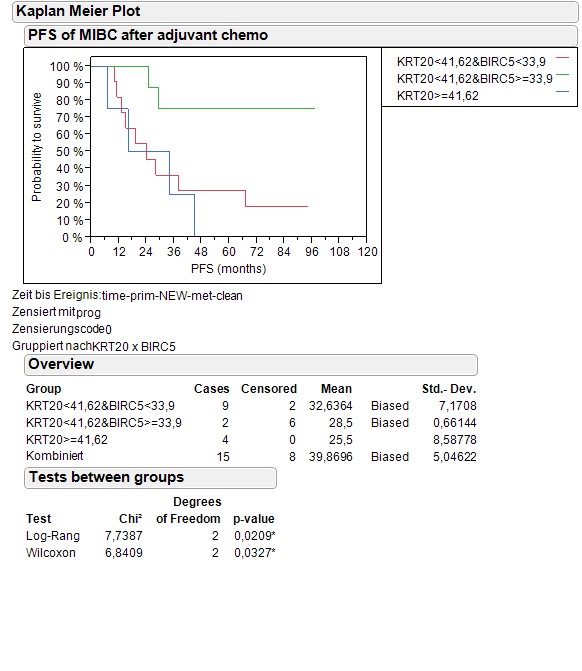

Supplement: Supplementary file 1 [file ijms-21-07420-s001.zip › supplementary figures Figure S1-S13/figS04.jpg]

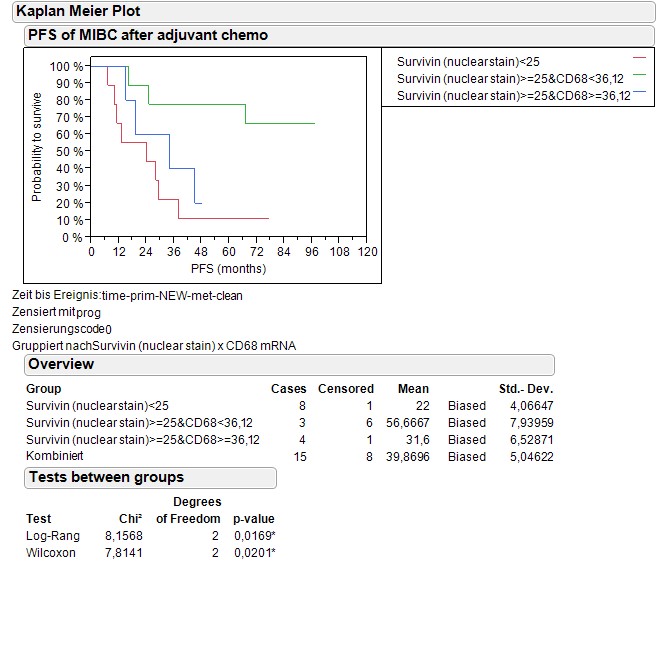

Supplement: Supplementary file 1 [file ijms-21-07420-s001.zip › supplementary figures Figure S1-S13/figS05.jpg]

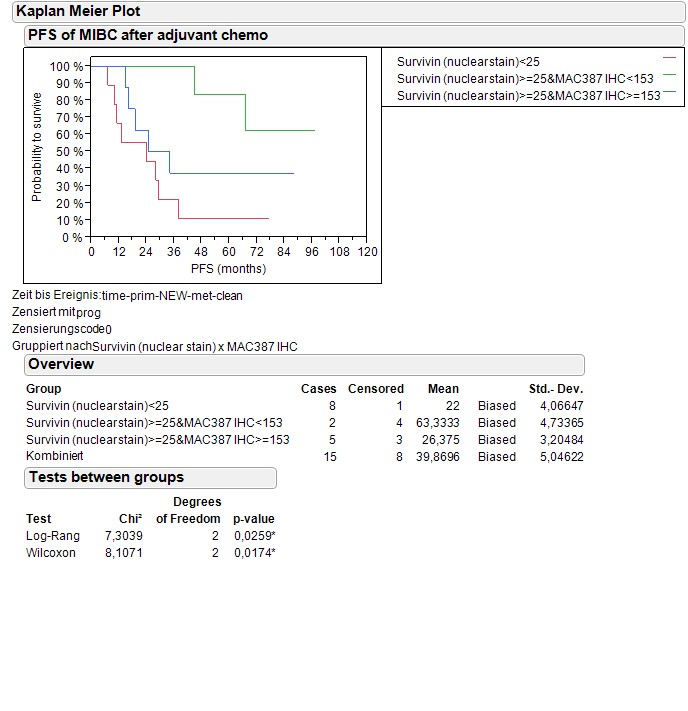

Supplement: Supplementary file 1 [file ijms-21-07420-s001.zip › supplementary figures Figure S1-S13/figS06.jpg]

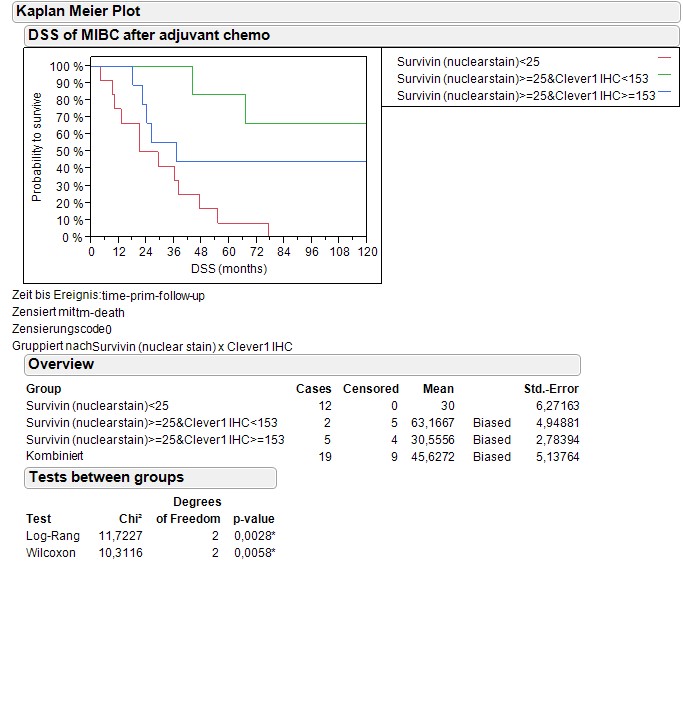

Supplement: Supplementary file 1 [file ijms-21-07420-s001.zip › supplementary figures Figure S1-S13/figS07.jpg]

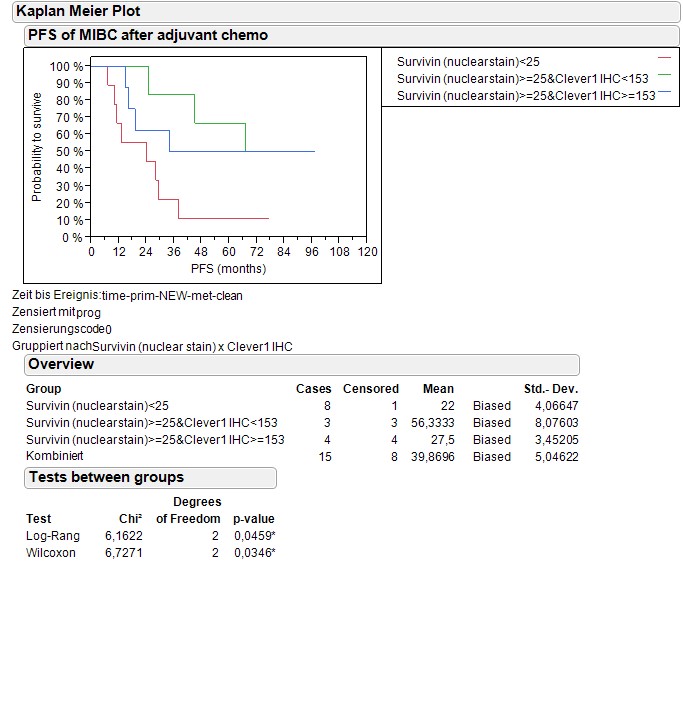

Supplement: Supplementary file 1 [file ijms-21-07420-s001.zip › supplementary figures Figure S1-S13/figS08.jpg]

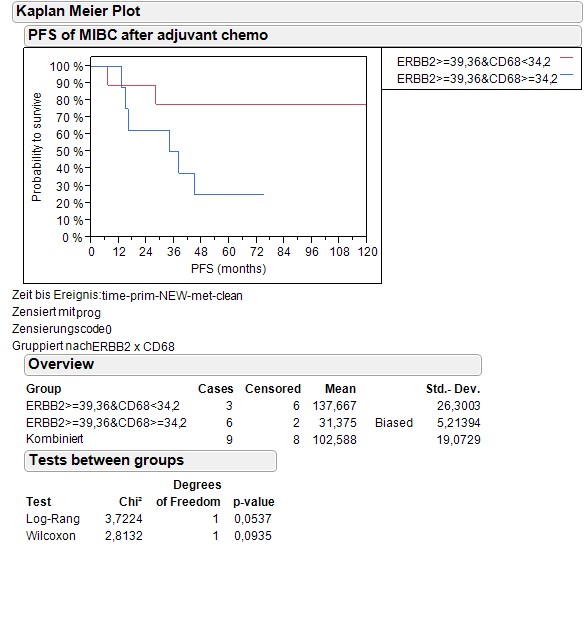

Supplement: Supplementary file 1 [file ijms-21-07420-s001.zip › supplementary figures Figure S1-S13/figS09.jpg]

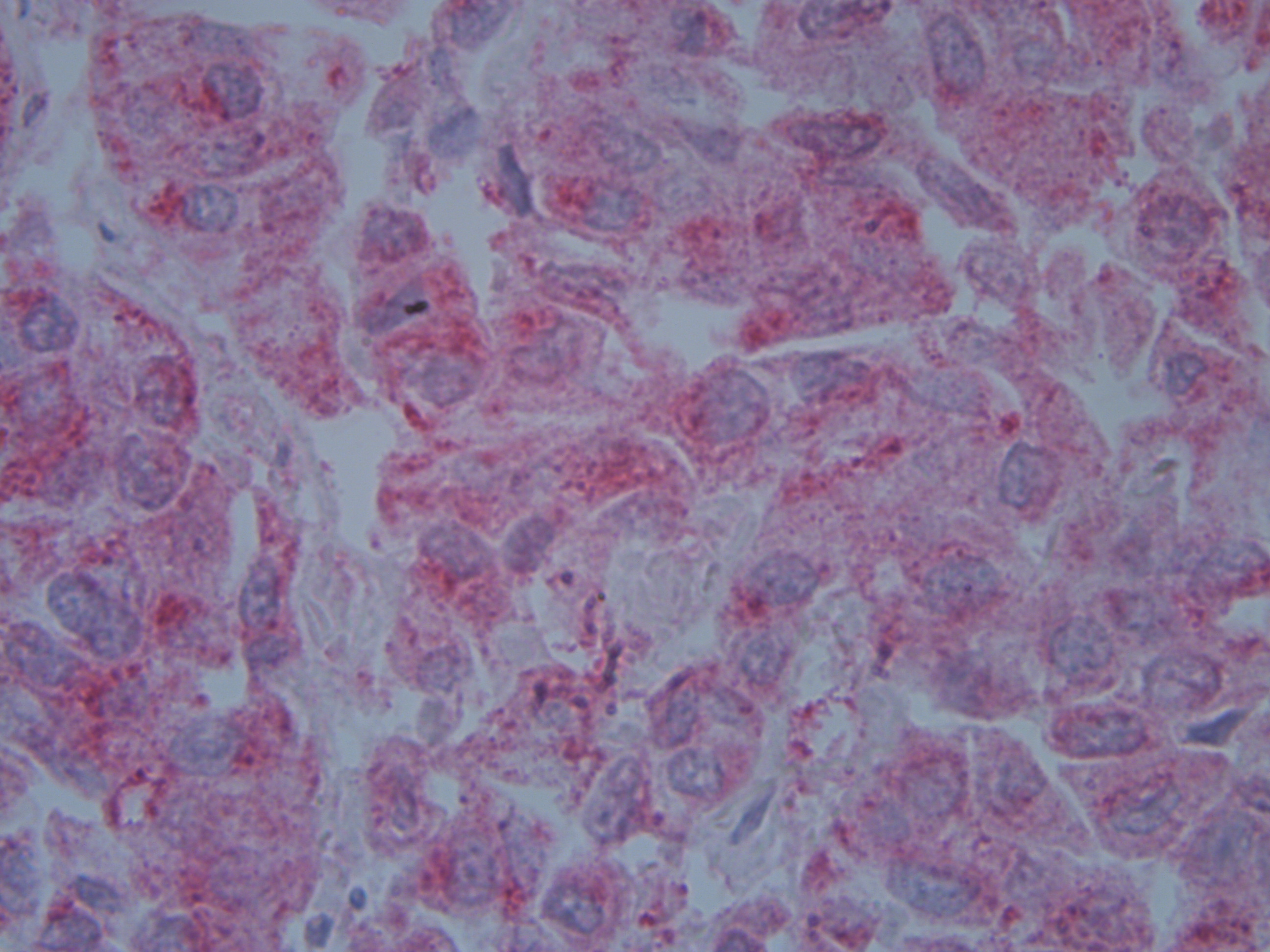

Supplement: Supplementary file 1 [file ijms-21-07420-s001.zip › supplementary figures Figure S1-S13/figS10.JPG]

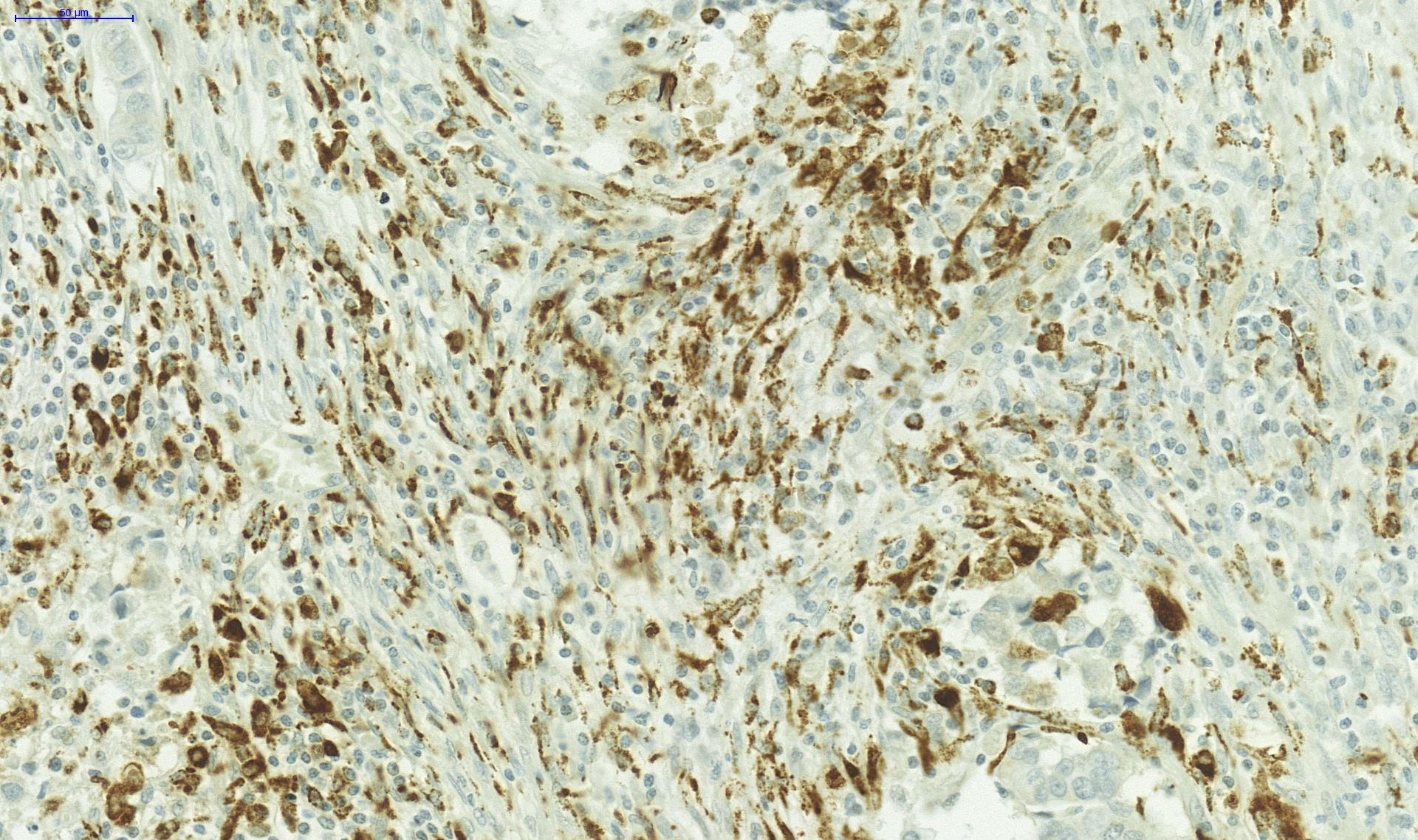

Supplement: Supplementary file 1 [file ijms-21-07420-s001.zip › supplementary figures Figure S1-S13/figS11.jpg]

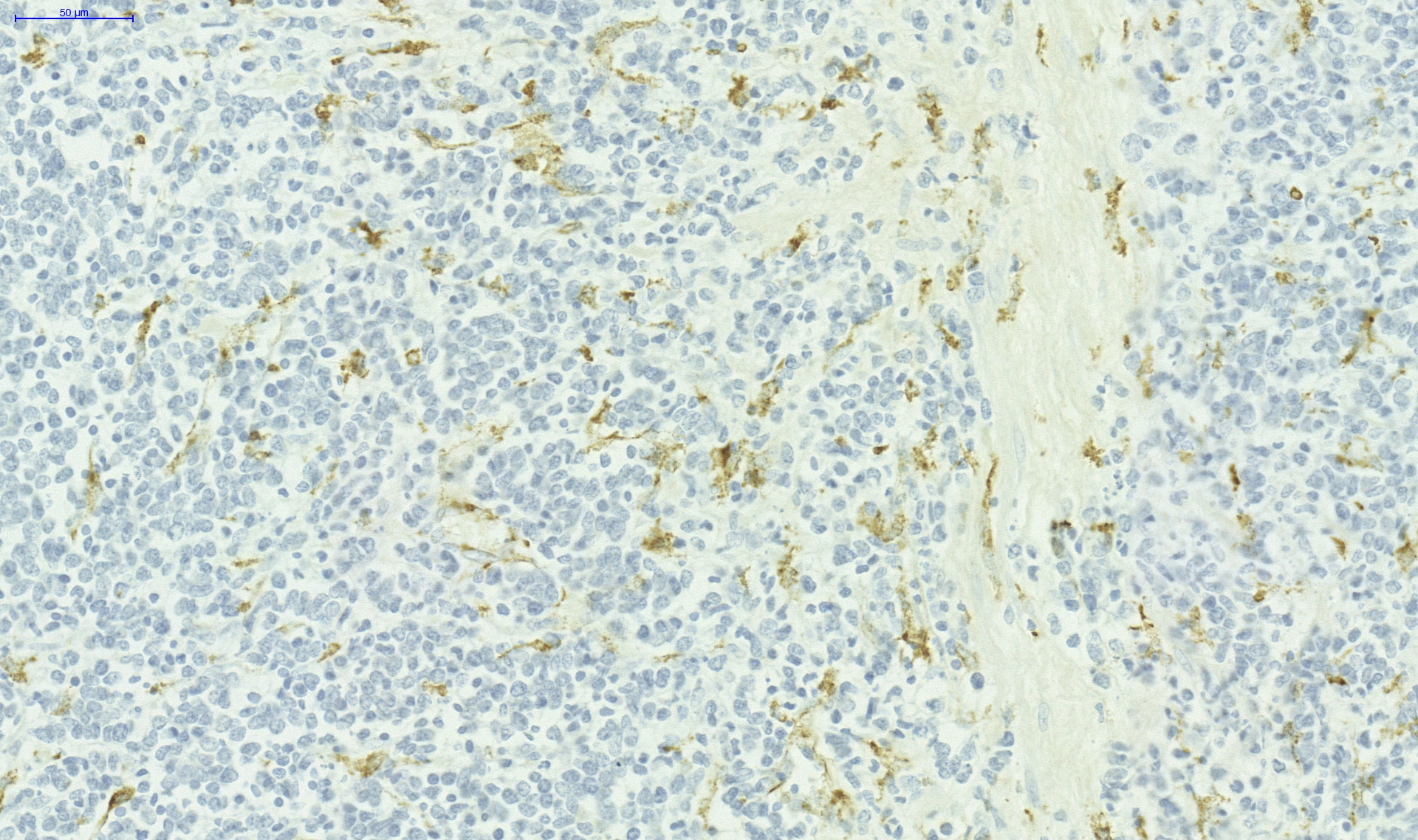

Supplement: Supplementary file 1 [file ijms-21-07420-s001.zip › supplementary figures Figure S1-S13/figS12.jpg]

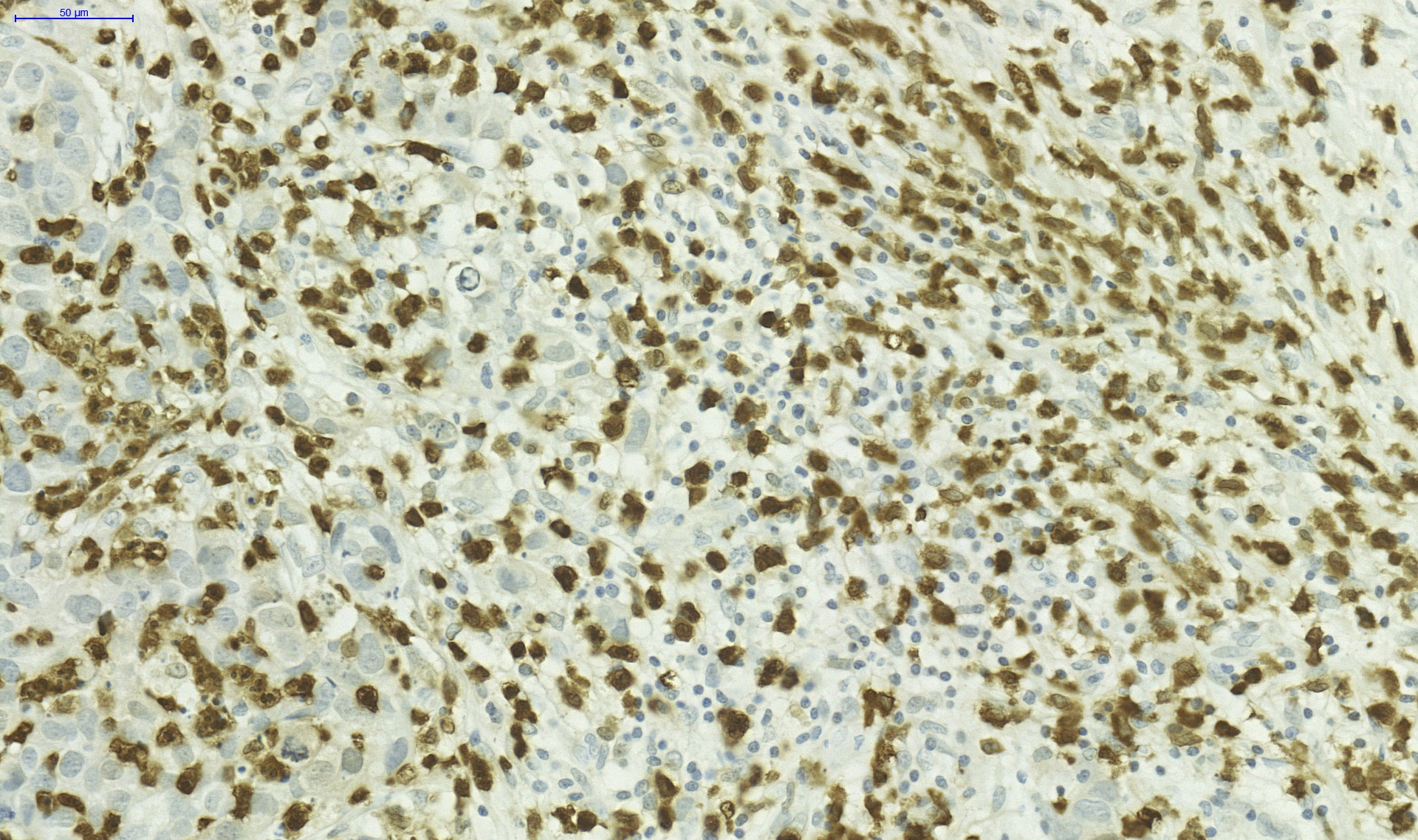

Supplement: Supplementary file 1 [file ijms-21-07420-s001.zip › supplementary figures Figure S1-S13/figS13.jpg]
